# No association between alcohol consumption and hip osteoarthritis: a diverse national analysis of 87,585 adults from the “All of Us” research program

**DOI:** 10.1007/s10067-026-08279-5

**Published:** 2026-07-11

**Authors:** Shlok V. Patel, Shujaa T. Khan, Smit Brahmbhatt, Matthew E. Deren, Nicolas S. Piuzzi

**Affiliations:** Department of Orthopaedic Surgery, Cleveland Clinic, Cleveland, OH 44195 USA

**Keywords:** Alcohol consumption, Hip osteoarthritis, Osteoarthritis, Risk factor

## Abstract

**Introduction:**

Hip osteoarthritis (OA) is estimated to affect 62.6 million individuals by 2050. A probable link exists between alcohol use and hip OA. However, the results are inconsistent, and the relationship between alcohol and hip OA remains speculative. To address these gaps, this study aimed to utilize the diverse, nationally representative All of Us Research Program dataset to explore the association between alcohol consumption and hip OA.

**Methods:**

This retrospective case–control study utilized data from the All of Us Research Program Controlled Tier Dataset v8. 17,517 hip OA cases and 70,068 controls were identified. A 1:4 case-to-control matching ratio was applied based on age and sex. Alcohol use frequency was categorized into five levels: Never, Monthly or Less, Two to Four Times per Month, Two to Three Times per Week, and Four or More Times per Week. Multivariable logistic regression models evaluated the association between alcohol use frequency and hip OA after adjusting for demographic and clinical variables.

**Results:**

Multivariable analysis found that alcohol use frequency was not significantly associated with hip OA. Compared to never users, participants with low (OR 0.98, 95% CI 0.93–1.04, *P* = 0.583), moderate (OR 0.99–1.01, all *P* > 0.05), and high (OR 1.02, 95% CI 0.95–1.09, *P* = 0.599) levels of alcohol consumption had no statistically significant differences in odds of hip OA. Female sex, Asian race, diabetes, hypertension, hyperlipidemia, and nicotine dependence increased the odds of hip OA.

**Conclusion:**

Any level of alcohol consumption was not significantly associated with the odds of hip OA. This study adds valuable insight to the current body of conflicting evidence. Further prospective studies appear warranted to shed light on the long-term effects of different alcoholic beverages on different joints.

**Table:** 

**Key Points** • *This study found no significant association between any degree of alcohol consumption and the odds of developing hip osteoarthritis*. • *Utilizing data from 87,585 adults in the NIH “All of Us" Research Program, this is the first study to analyze this relationship in a large, nationally representative population*. • *The research provides clarity to previously conflicting literature by demonstrating that alcohol lacks a clear harmful or protective effect on the clinical course of the disease*. • *The analysis highlights that independent risk factors such as Asian race, nicotine dependence, and components of metabolic syndrome increase the odds of hip osteoarthritis*.

## Introduction

Hip osteoarthritis (OA) is a common complex degenerative disease of the hip joint, with global cases projected to exceed 60 million by 2050 [1–3]. It is a leading cause of disability worldwide, with an increasing trend in incidence rates and disability-adjusted life years (DALYs) with the ageing of the population [2, 4]. The cause of hip OA is likely multifactorial, and all the major risk factors such as age, gender, BMI and genetics have been extensively researched [5–7]. However, the underlying cause and pathogenesis indicated in the development and progression of the disease remains widely unknown, largely because of an inability to account for confounding variables [8].

Upregulated inflammatory processes and oxidative stress to the articular cartilage contribute to the disease course of OA [9]. Alcohol consumption promotes the generation of reactive oxygen species and induces pro-inflammatory states [10]. Given these effects, it is biologically plausible that alcohol intake is a contributor to the pathogenesis of OA. Certain studies found alcohol to be associated with an increased risk of OA [8, 11, 12], whereas a few other studies reported no significant association between alcohol and OA, specifically hip OA [13–15]. Intriguingly, some studies have even proposed a protective effect of alcohol on inflammatory joint diseases [15–18].

The relationship between alcohol consumption and the development of OA is unclear at present. Current evidence is conflicting, and most studies have analyzed alcohol as a covariate while also lacking adequate sample sizes. This study attempts to utilize the diverse, nationally representative “All of Us Research Program” dataset and investigate the association between varying levels of alcohol intake and hip OA in a large sample of patients [19].

## Methods

### Study design

We conducted a retrospective, cross-sectional, case–control study utilizing data from the All of Us Research Program Controlled Tier Dataset v8, released in February 2024. The All of Us Research Program is a national precision medicine initiative that integrates electronic health records (EHR), survey responses, and demographic data from participants across the United States [19]. Individuals aged 18 years or older were included if they had complete information on alcohol use frequency, hip osteoarthritis (OA) status, and relevant demographic and clinical covariates.

### Case and control definition

**Fig. 1:**
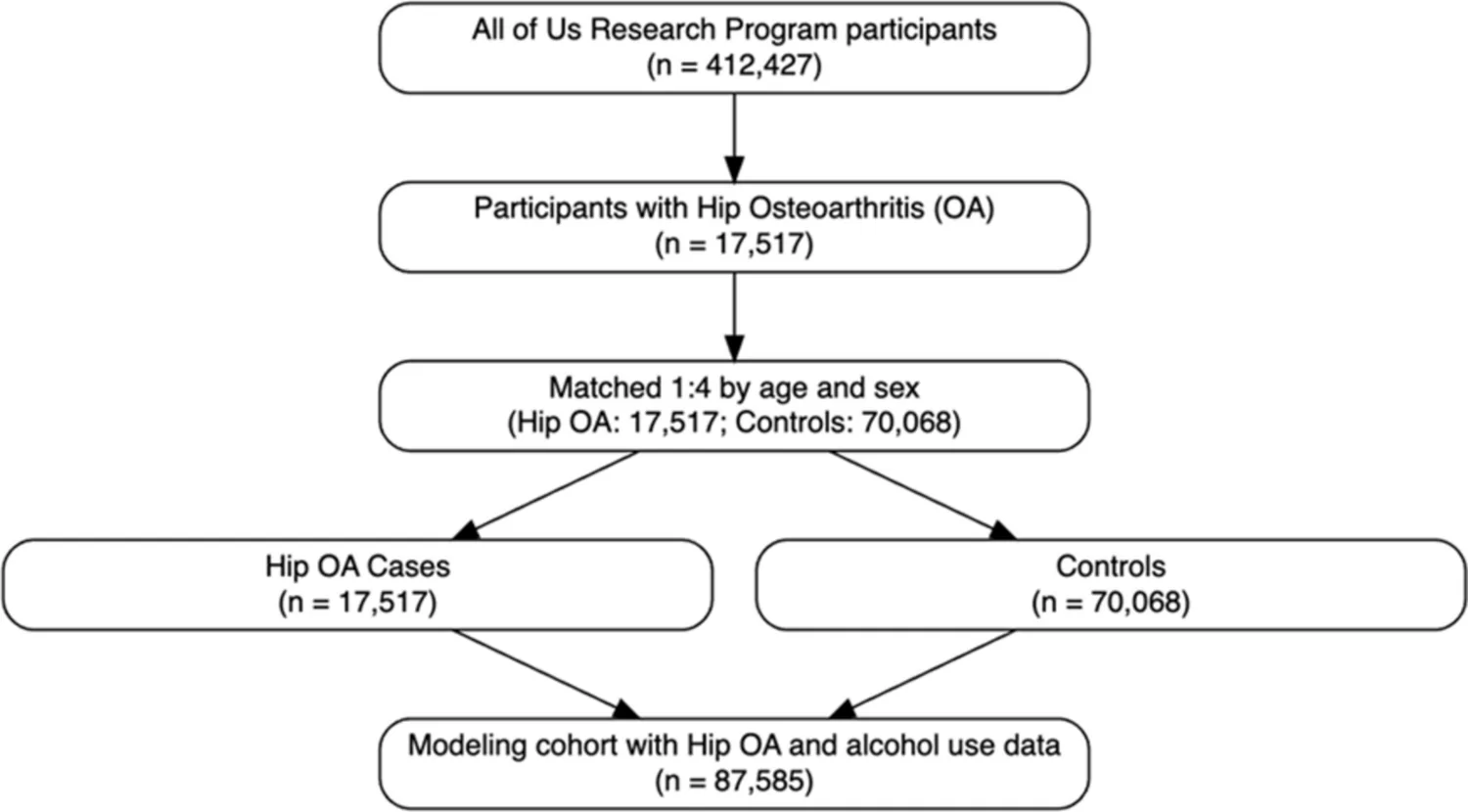
Flow diagram of cohort selection for hip osteoarthritis analysis. Participants diagnosed with hip osteoarthritis (*n* = 17,517) were matched 1:4 by age and sex to eligible controls (*n* = 70,068). After excluding individuals with missing alcohol use or covariate data, the final analytic cohort included 87,585 participants

Cases were defined as participants with at least one EHR-documented diagnosis of hip OA, identified through standardized SNOMED concepts mapped to ICD-9 and ICD-10 codes. Controls were defined as participants with no documented diagnosis of osteoarthritis in their EHR. To ensure computational efficiency while maintaining statistical power, we implemented a 1:4 case-to-control random sampling ratio, resulting in a final matched cohort of 87,585 individuals (17,517 hip OA cases and 70,068 controls), matched on age and sex (Fig. 1).

### Variables and covariates

Alcohol use frequency was self-reported via the All of Us Lifestyle survey in response to the question: “In the past year, how often did you drink any type of alcoholic beverage?” Responses were categorized into five levels: Never, Monthly or Less, Two to Four Times per Month, Two to Three Times per Week, and Four or More Times per Week. Participants with missing, ambiguous, or non-disclosed alcohol use data were excluded from the analysis. Covariates included age (continuous), gender identity, race, and ethnicity. Additional clinical variables incorporated into the model included diagnoses of type 2 diabetes mellitus, hypertension, hyperlipidemia, and nicotine dependence.

### Statistical analysis

We used multivariable logistic regression to evaluate the association between alcohol use frequency and hip OA case status. Alcohol frequency was modeled as a categorical variable, with “Never” serving as the reference group. Results were reported as adjusted odds ratios (ORs) with corresponding 95% confidence intervals (CI) and *P*-values. All statistical analyses were performed using R version 4.3.0 within the All of Us Researcher Workbench cloud-based environment.

Participants diagnosed with hip osteoarthritis (*n* = 17,517) were matched 1:4 by age and sex to eligible controls (*n* = 70,068). After excluding individuals with missing alcohol use or covariate data, the final analytic cohort included 87,585 participants.

## Results

A total of 87,585 participants with a median age of 72 years were included in the study. Majority of the participants were white (75%) and of female gender (62%). Alcohol intake varied according to OA status (*P* < 0.001); individuals with hip OA had a higher proportion of ‘never’ (22 vs 20.5%) and lower proportions of “2 to 4 per month”(18 vs 19%), “2 to 3 per week” (13 vs 14%), and “4 or More Per Week” (16 vs 17%) consumption categories compared to controls. Participants in the OA cohort had a higher prevalence of diabetes (22 vs 17%), hypertension (7.6 vs 5.5%), hyperlipidemia (89% vs 88%), and nicotine dependence (24 vs 19%) (*P*-values < 0.001) (Table 1).

**Table 1:** Participant characteristics by hip osteoarthritis status

		**Overall (*n* = 87,585)**	**Control (*n* = 70,068)**	**Hip OA (*n* = 17,517)**	***P*-value**
	Age (Median [IQR])	72 [64, 78]	72 [64, 78]	72 [64, 78]	0.926
**Gender**					0.338
	Female	54,564 (62%)	43,650 (62%)	10,914 (62%)	
	Gender identity: additional options	30 (< 0.1%)	21 (< 0.1%)	9 (< 0.1%)	
	Gender identity: non binary	103 (0.1%)	79 (0.1%)	24 (0.1%)	
	Gender identity: transgender	45 (< 0.1%)	34 (< 0.1%)	11 (< 0.1%)	
	Male	32,795 (37%)	26,250 (37%)	6545 (37%)	
	Not man only, not woman only, prefer not to answer, or skipped	48 (< 0.1%)	34 (< 0.1%)	14 (< 0.1%)	
**Race**					0.994
	American Indian or Alaska Native	683 (0.8%)	545 (0.8%)	138 (0.8%)	
	Asian	644 (0.7%)	515 (0.7%)	129 (0.7%)	
	Black or African American	15,516 (18%)	12,410 (18%)	3106 (18%)	
	Middle Eastern or North African	289 (0.3%)	231 (0.3%)	58 (0.3%)	
	More than one population	3572 (4.1%)	2853 (4.1%)	719 (4.1%)	
	Native Hawaiian or Other Pacific Islander	25 (< 0.1%)	18 (< 0.1%)	7 (< 0.1%)	
	White	65,845 (75%)	52,687 (75%)	13,158 (75%)	
**Ethnicity**					0.859
	Hispanic or Latino	1409 (1.6%)	1119 (1.6%)	290 (1.7%)	
	Not Hispanic or Latino	85,165 (97%)	68,140 (97%)	17,025 (97%)	
**Alcohol** u**se**					< 0.001
	Never	18,265 (21%)	14,385 (21%)	3880 (22%)	
	Monthly or less	26,328 (30%)	21,013 (30%)	5315 (30%)	
	2 to 4 per month	16,314 (19%)	13,101 (19%)	3213 (18%)	
	2 to 3 per week	12,135 (14%)	9779 (14%)	2356 (13%)	
	4 or more per week	14,543 (17%)	11,790 (17%)	2753 (16%)	
**Type 2** d**iabetes** m**ellitus**					< 0.001
	No	32,761 (82%)	22,210 (83%)	10,551 (78%)	
	Yes	7331 (18%)	4431 (17%)	2900 (22%)	
**Hypertensive** d**isorder**					< 0.001
	No	37,607 (94%)	25,176 (95%)	12,431 (92%)	
	Yes	2485 (6.2%)	1465 (5.5%)	1020 (7.6%)	
**Hyperlipidemia**					< 0.001
	No	4753 (12%)	3269 (12%)	1484 (11%)	
	Yes	35,339 (88%)	23,372 (88%)	11,967 (89%)	
**Nicotine** d**ependence**					< 0.001
	No	31,671 (79%)	21,502 (81%)	10,169 (76%)	
	Yes	8421 (21%)	5139 (19%)	3282 (24%)	

**Fig. 2:**
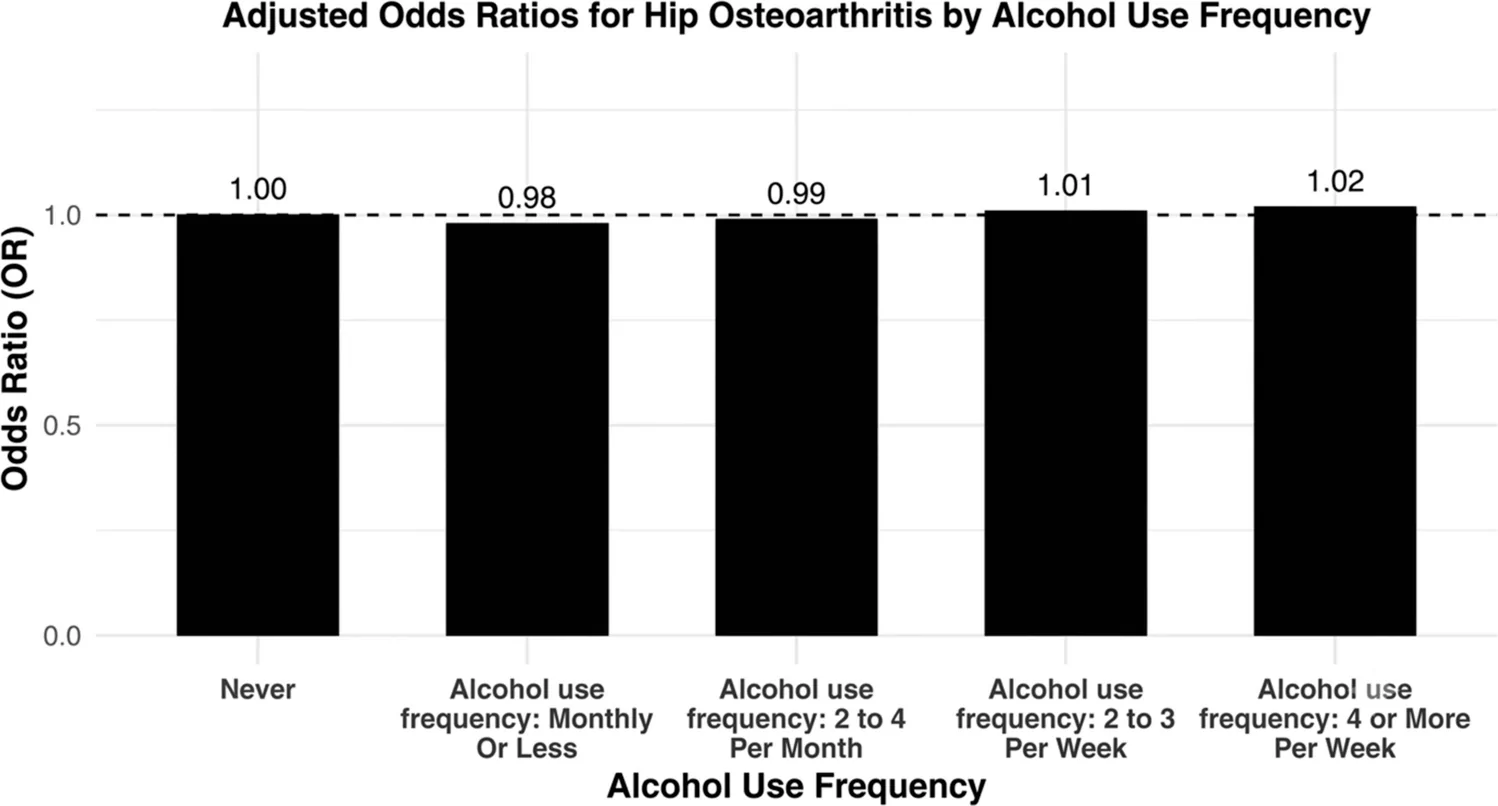
Adjusted odds ratios for hip osteoarthritis by alcohol use frequency. “Never” drinkers serve as the reference group (OR = 1.00). No statistically significant associations were observed across alcohol use frequency categories. Odds ratios remained close to 1, indicating no clear protective or harmful pattern

After adjusting for all demographic and clinical variables through multivariate logistic regression, alcohol use frequency was not significantly associated with hip OA. Participants with low (OR 0.98, 95% CI 0.93–1.04, *P* = 0.583), moderate (OR 0.99–1.01, all *P* > 0.05), and high (OR 1.02, 95% CI 0.95–1.09, *P* = 0.599) levels of alcohol consumption had no statistically significant differences in odds of hip OA compared to never users (Fig. 2). Both age (OR 0.99; *P* < 0.001) and male gender (OR 0.93; *P* = 0.002) were associated with lower odds of hip OA. Independent risk factors associated with increased odds of hip OA included Asian race (OR 1.44, 95% CI 1.01–2.06, *P* = 0.043), diabetes (OR 1.37, 95% CI 1.30–1.45, *P* < 0.001), hypertension (OR 1.42, 95% CI 1.31–1.55, *P* < 0.001), hyperlipidemia (OR 1.49, 95% CI 1.39–1.61, *P* < 0.001), and nicotine dependence (OR 1.47, 95% CI 1.38–1.55, *P* < 0.001) (Table 2).

**Table 2:** Multivariable Logistic Regression of Alcohol Use and Risk of hip osteoarthritis. Adjusted odds ratios (OR) and 95% confidence intervals (CI) from a logistic regression model evaluating the association between alcohol use frequency and hip OA status. Covariates include age, gender identity, race, ethnicity, diabetes, hypertension, hyperlipidemia, and nicotine dependence. “Never” is the reference group for alcohol use

Variable	OR (95% CI)	*P*-value
Alcohol use frequency: monthly or less	0.98 (0.93–1.04)	0.583
Alcohol use frequency: 2 to 4 per month	0.99 (0.92–1.05)	0.688
Alcohol use frequency: 2 to 3 per week	1.01 (0.94–1.09)	0.726
Alcohol use frequency: 4 or more per week	1.02 (0.95–1.09)	0.599
Age	0.99 (0.99–0.99)	** < 0.001**
Gender: additional options	2.25 (0.74–7.04)	0.147
Gender: non binary	1.61 (0.76–3.38)	0.204
Gender: transgender	1.93 (0.77–4.89)	0.156
Gender: male	0.93 (0.89–0.98)	**0.002**
Gender: not disclosed	6.32 (1.96–28.05)	**0.005**
Asian race	1.44 (1.01–2.06)	**0.043**
Black or African American race	1.04 (0.82–1.32)	0.776
Middle Eastern or North African race	1.06 (0.68–1.62)	0.797
More than one population race	1.06 (0.83–1.37)	0.645
Native Hawaiian or Other Pacific Islander race	1.67 (0.57–4.83)	0.339
None of the races listed	1.06 (0.76–1.47)	0.74
Not Hispanic or Latino ethnicity	0.90 (0.75–1.07)	0.227
Diabetes	1.37 (1.30–1.45)	** < 0.001**
Hypertension	1.42 (1.31–1.55)	** < 0.001**
Hyperlipidemia	1.49 (1.39–1.61)	** < 0.001**
Nicotine dependence	1.47 (1.38–1.55)	** < 0.001**

Odds ratios (OR) with 95% confidence intervals (CI) estimated from multivariable logistic regression. Outcome variable: diagnosis of hip osteoarthritis (yes vs. no). Alcohol use frequency is modeled as a categorical exposure with “Never” as the reference group. Models are adjusted for all listed covariates. Bold *P*-values indicate statistical significance (*P* < 0.05). Reference categories for covariates: female sex, White race, Hispanic or Latino ethnicity, and no comorbidities

## Discussion

OA is the most common joint pathology leading to disability, pain, and worsened quality of life [1, 20]. Despite its high global burden, there is no established strategy to either prevent the disease or modify its progression [21]. In this regard, this study aimed to analyze the role of alcohol intake as a modifiable factor in the development and progression of hip OA. This study utilized the NIH’s *All of Us* Research Program dataset, which is a longitudinal cohort study aimed at advancing precision medicine through partnering with one million or more diverse participants across the United States [19]. To the best of our knowledge, this is the first study to analyze the role of alcohol consumption in the development of hip OA in a large, nationally representative patient population.

Our multivariable analysis reported no significant associations across all the categories of alcohol use frequency. Other independent risk factors of hip OA like female gender, Asian race, diabetes, hypertension, hyperlipidemia, and nicotine dependence were identified. These data suggest that alcohol may not have a significant harmful or protective effect on the hip joint and consequently, may not be contributing towards the disease course of hip OA. The results of this study offer much needed clarification given the contradictory existing evidence. They also help advance the understanding of the etiology of hip OA and aid in the development of predictive models for identifying high-risk populations.

OA is characterized by cartilage loss, synovial inflammation, and formation of osteophytes with an increase in C-reactive protein and reactive oxygen species, indicating augmented inflammatory processes in the joint [21]. Long-term and excessive alcohol consumption causes raised blood levels of inflammatory mediators. It also has detrimental effects on the musculoskeletal system such as increased risks of osteoporosis and myopathies [5]. This theory suggests the potential involvement of alcohol in the disease course of OA. Numerous studies add weight to this theory, starting with an in-vivo experimental model study, which concluded that chronic alcohol intake induces severe arthritic changes in both knee and shoulder joints of mice [8]. Another study in around 83,400 women found a dose-dependent association of alcohol consumption with the incidence of THA due to hip OA [21]. A case–control study reported beer consumption as a risk factor of knee and hip OA [15]. There is another school of thought wherein alcohol intake specifically, low-to-moderate, is protective against OA [22]. This stems from the theory that low dose persistent ethanol consumption interferes with NF-KB mediated mechanisms and downregulates production of pro-inflammatory mediators and leukocyte migration [18]. Literature suggesting the paradoxically beneficial effects of alcohol on multiple inflammatory diseases such as RA, axial spondyloarthritis, and SLE provides further validation to this conjecture [23–25]. Also, one study found wine consumption to be negatively associated with knee OA, and spirit consumption to be negatively associated with hip OA [15]. Further, another study found that alcoholic beverage, especially beer, has a protective effect on knee OA, with patients who consumed > 0.6 g/day showed less knee OA than those who consumed < 0.6 g/day [22]. While these findings highlight both the hazardous and beneficial effects of alcohol consumption with respect to OA, it is worth noting that several other studies have found no association between them, offering a more nuanced perspective on its overall impact. For instance, a meta-analysis of 29 studies and 25,192 patients investigated the relationship between alcohol and osteoarthritis [16]. Although it found a negative association of weekly or more frequent alcohol intake with OA, the association vanished after adjusting for confounding variables.

Existing evidence on the effect of alcohol in OA is inconsistent and inconclusive. This could be due to heterogeneity in studies, lack of adjustment for covariates, small sample sizes, variability in diagnostic criteria of OA, irregularities in the joint studied, and inability to account for differences in the frequency and type of alcoholic beverage consumed. In this study, crude analyses demonstrated a statistically significant negative association of hip OA with the frequency of alcohol consumption. However, in line with the results of the meta-analyses, the association became insignificant following adjustment for demographic and clinical variables [16]. Our study provides evidence that alcohol is unlikely to play a significant role in the pathophysiology of hip OA. Another nationwide large-scale study in the Korean population was also unable to document a significant association between radiologically diagnosed hip OA and alcohol, thereby corroborating our findings [5]. On the other hand, one of the largest studies to date in women with a 32-year follow-up period, found a dose-dependent increase in alcohol mediated risk of THA due to hip OA [21]. This held true for all alcohol types and the heterogeneity based on alcohol type was statistically insignificant. While this contradicts the results of our study, a possible reason could be our inability to segregate the analysis based on gender. Additionally, the study only included the severe end-stage symptomatic hip OA which could have altered the findings. A case–control study in the UK analyzed the effects of different types of alcohol consumption with OA. It was found that a total alcohol intake of < or = 2 servings a week, as well as spirit consumption of 4–8 servings a week or > or = 8 servings a week, was protective against hip OA. But beer intake of 8–19 per week or > or = 20 per week was a significant risk factor for hip OA, with a dose–response association [15]. Wine was not associated with hip OA. The variation of effect based on alcohol type offers an alternative theory suggesting that alcohol itself may not be responsible for influencing the disease progression of hip OA, instead other factors within spirits, wine, and beer may explain their differential associations with OA. This could explain the conflicting evidence investigating the effects of alcohol on OA. Keeping that in mind, the absence of any significant association between alcohol and hip OA in our study can change if individual alcohol type is accounted for. Our adjusted analyses noted age and male gender to be negatively associated with the odds of hip OA. It is a well-known fact that the incidence of OA increases with older age [13, 26]. The negative association of age despite a higher unadjusted prevalence of older individuals potentially reflects residual confounding or survivor bias. The negative correlation of OA with male gender is an established fact. As reported by previous studies, female gender is generally at a higher risk, particularly after menopausal age [27]. Our study found Asian race as an individual risk factor for hip OA. This contrasts existing literature that reports a lower prevalence of hip OA in the Asian population compared to the whites [28]. This disparity could be due to the prior studies primarily examining the Asian population in their native countries. Our study however, extracted data from a large, nationally representative, diverse American population, which facilitated the study of racial minorities within a shared healthcare system and environment. This subgroup result should nonetheless be interpreted cautiously, as it was derived from a small number of Asian participants. Several mechanisms could plausibly contribute to a higher recorded burden of hip OA in this group, including a higher prevalence of acetabular dysplasia and other subtle developmental variants of hip morphology, which are well-recognized precursors of secondary hip OA [29]. Other individual risk factors noted by our analyses were diabetes, hypertension, and hyperlipidemia. This aligns with the existing research which states a strong positive association between metabolic syndrome and OA, with the risk of OA increasing following the addition of each component of metabolic syndrome [30, 31]. It is important to note that, even though our study concluded that alcohol is not a risk factor for hip OA, it may still indirectly affect joint health. Studies have shown that alcohol increases the likelihood of metabolic syndrome, hypertension, and dyslipidemia, all of which may contribute to an elevated risk of hip OA [32, 33]. Nicotine dependence was independently associated with higher adjusted odds of hip OA in the present study. This finding should be interpreted in the context of mixed prior literature and the complex, dose-dependent relationship between nicotine, tobacco exposure, and cartilage biology. Prior epidemiologic studies have reported an apparent protective association between smoking and radiographic knee or hip OA, and experimental studies have suggested that nicotine at lower or physiologic concentrations may promote chondrocyte proliferation, glycosaminoglycan synthesis, and type II collagen synthesis [34–37]. However, higher or sustained nicotine exposure has been associated with suppression of chondrocytes and chondrogenic markers, including Sox9, type II collagen, and aggrecan [38, 39]. In addition, cigarette smoke contains numerous non-nicotine toxic constituents that may promote oxidative stress, chondrocyte injury, and cartilage damage [40]. Therefore, the positive association between nicotine dependence and hip OA observed in our study may reflect a heavier or more chronic tobacco-exposure phenotype.

The strengths of this study include a large sample size, nationally representative cohort, and adjusted model including multiple demographic and clinical variables. Prior validation work has shown that the All of Us Researcher Workbench integrates survey, physical measurement, and EHR data and can reproduce known clinical associations [41]. In addition, prior All of Us studies using alcohol-related survey data have demonstrated clinically coherent associations with liver injury and hazardous drinking patterns [42, 43]. Within the present model, established clinical correlates of hip OA, including diabetes, hypertension, hyperlipidemia, and nicotine dependence, were also associated with hip OA in the expected direction, supporting the internal face validity of the dataset and analytic model. This study adds meaningful findings to the pool of literature on the etiology and risk factors of hip OA. The study findings should inform clinicians on potential prevention strategies and predictive models focusing on other modifiable risk factors that have stronger evidence of contributing to hip OA. The limitations of this study encompass the retrospective design and lack of analysis by alcohol type. Additionally, alcohol use was documented only for the past year, thereby factoring out the effects of cumulative alcohol use. Furthermore, some individuals with OA may deviate from their usual alcohol consumption, for example to self-medicate for pain. Finally, alcohol consumption was self-reported, which may introduce some recall bias or incorrect reporting of actual intake due to potential social stigma. These factors could skew the results of this study. Considering the non-uniformity of results from previous studies, further prospective cohort studies should be undertaken to uncover the association between alcoholic beverages and hip OA. These studies should account for cumulative alcohol use, type of alcohol consumed, type of joint, other confounders, and effect modifiers.

## Conclusions

In this large, nationally representative study, we found no significant association between any degree of alcohol consumption and the odds of hip OA; therefore, there is no clear protective or harmful effect of alcohol on the clinical course of hip OA. These findings provide clarity to a previously conflicting body of literature. However, the relationship between alcohol and hip OA remains speculative. We observed associations between hip OA and metabolic syndrome, which emphasizes the value of metabolic health in OA risk stratification. Therefore, while alcohol itself may not significantly influence the course of hip OA, its indirect effects via modifiable comorbidities should be examined. Also, further prospective research, including but not limited to the role of different alcohol beverages and the cumulative effects of their long-term consumption on different joints, should be conducted to provide more clarity. Until such evidence is available, focus should be placed on established modifiable risk factors for prevention of OA in the hip joint.

## Data Availability

The dataset is securely available to approved investigators via the NIH All of Us Research Program platform.
